# A prediction model for COPD readmissions: catching up, catching our breath, and improving a national problem

**DOI:** 10.3402/jchimp.v2i1.9915

**Published:** 2012-04-30

**Authors:** Bravein Amalakuhan, Lukasz Kiljanek, Arvin Parvathaneni, Michael Hester, Pramil Cheriyath, Daniel Fischman

**Affiliations:** Department of Internal Medicine, Pinnacle Health System-Harrisburg Hospital, Harrisburg, PA, USA

**Keywords:** COPD, random forest, prediction, readmission

## Abstract

Frequent COPD exacerbations have a large impact on morbidity, mortality and health-care expenditures. By 2020, the World Health Organization expects COPD and COPD exacerbations to be the third leading cause of death world-wide. Furthermore, In 2005 it was estimated that COPD exacerbations cost the U.S. health-care system 38 billion dollars. Studies attempting to determine factors related to COPD readmissions are still very limited. Moreover, few have used a organized machine-learning, sensitivity analysis approach, such as a Random Forest (RF) statistical model, to analyze this problem. This study utilized the RF machine learning algorithm to determine factors that predict risk for multiple COPD exacerbations in a single year.

This was a retrospective study with a data set of 106 patients. These patients were divided randomly into training (80%) and validating (20%) data-sets, 100 times, using approximately sixty variables intially, which in prior studies had been found to be associated with patient readmission for COPD exacerbation. In an interactive manner, an RF model was created using the training set and validated on the testing dataset. Mean area-under-curve (AUC) statistics, sensitivity, specificity, and negative/positive predictive values (NPV, PPV) were calculated for the 100 runs.

The following variables were found to be important predictors of patients having at least two COPD exacerbations within one year: employment, body mass index, number of previous surgeries, administration of azithromycin/ceftriaxone/moxifloxacin, and admission albumin level. The mean AUC was 0.72, sensitivity of 0.75, specificity of 0.56, PPV of 0.7 and NPV of 0.63. Histograms were used to confirm consistent accuracy.

The RF design has consistently demonstrated encouraging results. We expect to validate our results on new patient groups and improve accuracy by increasing our training dataset. We hope that identifying patients at risk for frequent readmissions will improve patient outcome and save valuable hospital resources.

Chronic obstructive pulmonary disease (COPD) is a major cause of morbidity and mortality worldwide. To understand the gravity of this problem, by 2020, the World Health Organization expects COPD to be the third leading cause of death in the United States (1, 2). Furthermore, in 2002, it was estimated that COPD and its related complications cost the US health care system 32 billion US dollars, and this burden is likely to rise beyond the year 2025 (3). In addition, COPD is the only major disease with an increasing death rate, rising at 16% per year (4). Given the individual, social, and economic impact of COPD morbidity, studies have begun to delineate some of the factors related to recurrent COPD exacerbations. But, these studies are still very limited, mainly due to a lack of publicity and attention focused on this condition.

Considering the increasing societal burden of COPD, new and innovative ways to minimize the societal toll it exacts are needed. One approach that may be employed to identify critical factors related to COPD and its comorbidities is to utilize machine-learning algorithms to determine where our limited societal resources may be most effectively deployed. One such machine-learning algorithm that has consistently shown accuracy in critical risk factor identification is the random-forest (RF) statistical model (5–8). This model is slowly becoming a staple in the world of higher-level statistical analyses, as well as becoming an adjunct to classical data analysis of complex problems (6, 8). The RF model has already been used by the National Institutes of Health (NIH) with consistent success in the areas of cellular and molecular biology, genetics, and pharmacology (7, 9, 10). Moreover, a recent study reported in the *Journal of Artificial Intelligence Medicine* (2008) compared eight of the most robust mathematical prediction models known; the RF model consistently had the best results (5).

Thus, the objective of our study is to employ the RF statistical model to determine factors highly correlated with COPD-related hospital readmissions and create a computer algorithm that can predict which patients are at high risk for hospital readmission within the year following the index admission. As a result of this risk factor analysis, earlier and more aggressive goal-directed therapy could be judiciously utilized in order to improve patient outcome, resulting in more cost-effective utilization of limited medical resources.

## Methodology

### Definitions

‘Index admission (IA)’ was defined as the first admission in a consecutive 12-month time period. The 12-month period was determined from the IA forward, with the IA being considered time zero. This IA was the admission period, where data were gathered from and then used to predict subsequent readmissions.

‘Frequent or multiple COPD readmissions/exacerbations’ were defined as two or more readmissions within a consecutive 12-month period, beginning with the date of IA.

‘Regression Trees’ are binary trees with nodes that correspond to different values in the input variables. These trees are developed using a training set. At each node or branch/partition, the RF algorithm searches for a value that best separates all instances within that node based on the outcome of interest. If the value chosen is able to classify all instances so that all instances are in one of the two descendent branches/nodes, then this node becomes a terminal node. If not, this process is repeated until all instances are at terminal nodes.

### Selection of variables for data collection

Based on a survey of current literature, 55 patient-specific variables, which have been found to be highly associated with COPD exacerbations, were identified (Tables 1 and 2). These variables were chosen only after they were found to have consistent and strong association with this study's outcome of interest, over many studies. In other words, only strongly validated variables were used. Variables with weak or mixed associations to COPD exacerbations were not used in this study to avoid creating a weak prediction algorithm.

**Table 1:** General variables collected

Age	BMI	History of CAD	History of diastolic heart failure
Sex	Marital status	History of NSTEMI	History of previous pneumonia
Race	Number of allergies	History of STEMI	History of asthma
History of drinking	Number of meds	History of HTN	Inhaled anticholinergic use
Living arrangement	Number of major diseases in past medical history	History of DM	Home oxygen use
Employment status	Number of surgeries in past medical history	History of systolic heart failure	Home B2 agonists use
Zip code of home	History of smoking	Most recent HbA1c	Up-to-date on influenza and pneumovax vaccine

**Table 2:** Variables collected within ‘index of admission (IA)/hospital stay’

Admission 02 saturation	Moxifloxacin use	HC03 on basic metabolic profile at discharge date	Month of admission
Lowest 02 saturation	PO/IV steroid use	Serum albumin on admission	Pneumonia
Beta-2 agonist use	PO steroid prescription upon discharge	Serum magnesium on admission	Length of stay in days
Inhaled anticholinergic use	pH, PaCO_2_, PaO_2,_ HC03, 02 saturation (ABG) a) on admission b) nearest to discharge date	Pulmonologist consulted	
Inhaled steroid use	HC03 on basic metabolic profile at admission date	Intubated	

### Sample selection

#### Sample collection

A retrospective review of medical records was performed on patients who were diagnosed with acute COPD exacerbations on admission based on International classification of disease (ICD)-9 codes. The selection of the study population, exclusion criteria, and sampling process were performed in compliance with standing policies and procedures of the Institutional Review Board.

*Inclusion criterion*. Patients who were diagnosed with a COPD exacerbation between January, 2007 and September, 2010.

*Exclusion criteria*. (1) Age less than 18 years; (2) Pregnancy.

#### Sample size

Data were collected for 106 patients. The prevalence of patients within our sample that had multiple readmissions within a single year was 0.47 (50 patients).

### Randomization and data analysis

Randomization and data analysis were performed using RF, receiver operating characteristic curve (ROC), and Caret programming packages (11, 12). The selected population was randomly divided into two separate data sets, 75% of the patients were assigned to the training data set (the algorithm creation group) and the remaining 25% were assigned to the testing data set (validation group). The training group was used to form the algorithm composed of 1,000 regression trees, with each tree a voting contributor to the mathematical prediction model. The testing/validation group was used to determine which arm of the tree was the ‘multiple readmissions’ arm and which was the ‘no readmission’ arm. In essence, this process was used to determine the most sensitive split of the variables, within each tree for the outcome of readmission. The aggregate of the 1,000 votes/predictions helped to create a probability of the patient being readmitted within a single year.

The training group and the testing/validation groups within the RF statistical method deserve further clarification. The RF statistical design uses 25% of the whole data set to create multiple validation groups, in an iterative fashion. The ‘25%’ figure only describes the size of one single validation group. In fact, the RF allows the inclusion of all the sample participants when all the data runs are completed. The same holds true for the training group, whereby multiple groups of 75% were sampled and the data/values from their collected variables were used to build a series of 1,000 regression trees. Furthermore, this partition of 75:25 has been shown in previous literature investigating the RF statistical design to be valid and to be an acceptable validation group size, despite only including 25% of the sample (11, 12).

Within each of these two groups, there was an equal ratio of patients with multiple readmissions to patients with no readmissions. The above process was repeated iteratively, 200 times. For each of these 200 runs, the following measures of accuracy were calculated to determine the predictive value of the model: (1) area under the curve (AUC) for ROC, (2) specificity, (3) sensitivity, (4) positive predictive value (PPV), and (5) negative predictive value (NPV). The statistics of accuracy for each of the 200 runs of the prediction model were then used on a distribution curve to determine the consistency of the results. The metric used to determine the ‘variables of importance’ was the highest AUC.

## Results

### Variables of importance

The following five variables were found to be important predictors of patients having at least two COPD exacerbations within one year: (1) employment status; (2) body mass index (BMI); (3) number of previous surgeries; (4) index of admission albumin level; and (5) whether there was administration of Azithromycin with Ceftriaxone during the IA.

### Accuracy of the prediction model

**Fig. 1:**
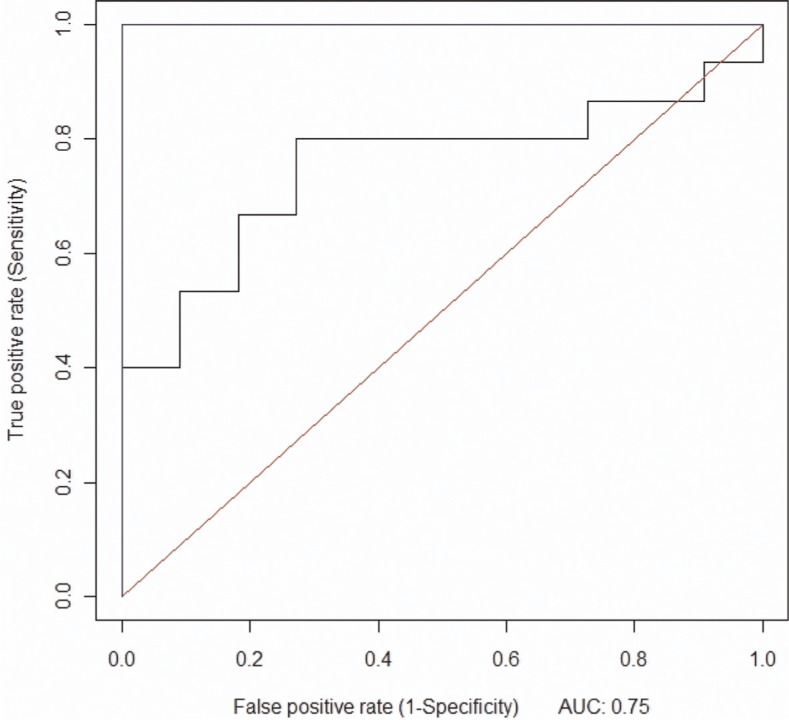
ROC curve generated in the final data run, using the random forest algorithm.

For the 200 runs of the prediction model, the mean AUC was 0.75, the sensitivity was 0.74, the specificity was 0.58, the PPV was 0.71, and the NPV was 0.63. Histograms were used to confirm consistency of the model's results. Please see Fig.1 for the ROC curve.

## Discussion

For decades, we have been struggling to contain this national health epidemic. Classical data analyses have been used to determine factors correlated with COPD exacerbations in the past, but these methods have not yielded results that have been able to curb the increase in morbidity and mortality related to COPD exacerbations (13–17). The reality is that new and innovative approaches are needed in the light of COPD's worrisome growth in morbidity and mortality. Considering the success that the RF predictive tool has had in other scientific disciplines, it would be anticipated to yield similar results when applied to the problem of COPD morbidity (5–10). Indeed, our results show that the RF analytical approach does provide useful information, from which early intervention programs may be developed to prevent readmissions related to COPD exacerbations.

The five variables identified in this study's prediction model illustrate that physicians should not only evaluate patients with COPD from a biological and chemical perspective, using lab values and imaging studies, but must also evaluate patients as a whole, taking into consideration epidemiological factors, social factors, as well as nutritional status. Viegi et al. in an extensive thematic review emphasized the impact on COPD of so-called social factors, including low-socioeconomic status, housing conditions, and diet (18).

Consistent with the findings of Viegi's review, one of the most sensitive variables found in this study was employment status. For this variable, patients were divided into five, mutually-exclusive categories: (1) employed; (2) unemployed; (3) retired (4); disabled; or (5) homemaker. It is likely that having employment was correlated with better access to health care. Several studies have also shown a consistent association of exercise with reduced COPD exacerbations (19). This would likely explain why patients with mobility challenges were also at increased risk for readmissions. Considering the importance of employment and thus socioeconomic status discovered in this study, an important intervention would be to have the early involvement of social workers in patient care. The goal of these social workers would be to connect patients with services in the community to help them obtain steady employment and other forms of economic support. This would likely improve their access to health care resources and more sanitized living conditions.

Like employment status, BMI and admission albumin level were highly predictive of COPD readmissions. These two indices are likely surrogate markers of nutritional status (20, 21). The strong association of COPD readmissions with BMI and serum albumin levels has been supported in previous studies (20, 22, 23). Furthermore, prior studies have also shown increased COPD related mortality rates in patients with a low BMI, again lending support to this study's findings (24, 25). Many patients with COPD become short of breath even when eating moderate-sized meals and as a result tend to eat less and become malnourished (26, 27). Considering so much of their energy and caloric intake is simply consumed by the work of breathing, malnutrition can result in ‘respiratory fatigue’ and leave patients susceptible to repeated COPD exacerbations (26). Thus, patients with COPD need to be educated on the importance of eating small yet more frequent meals. On the opposite end of the spectrum, an elevated BMI has been shown to be highly associated with obstructive sleep apnea, which in turn also impacts effective management of COPD and thus susceptibility to COPD exacerbations (28). Regardless of whether a patient has an elevated BMI or whether they are malnourished, the over-arching foundation of these variables seems to be the need for a ‘healthy nutritional status’. For these reasons, dietitians need to be involved in patient care, providing education on effective eating habits, as well as exercise.

Akin to a patient's nutritional status, the number of previous surgeries a patient has undergone can also impact overall health, potentially through its effects on the muscles of respiration. To our knowledge, no other study has reported increased risk of COPD readmissions in patients with an extensive surgical history; probably because it has not been analyzed in previous studies. One possible explanation for this finding is that an extensive surgical history may be a marker for increased risk of physiologically significant atelectasis, with resultant increase in oxygen demand and labored breathing (29). Furthermore, postoperative pain, especially with cardiothoracic and abdominal procedures decreases tidal volumes and leaves patients susceptible to hypoxia and hypercapnic respiratory failure (30, 31). The implication is that aggressive respiratory rehabilitation needs to be instituted for all postoperative patients with COPD (32).

Respiratory infections are a frequently reported cause of COPD exacerbations (33). This is likely the reason why the use of antibiotics has been consistently shown in literature to reduce COPD exacerbation rates (33–38). In this study, two antibiotic regimes were included in the RF analysis to delineate whether they had differing values in the prediction of COPD readmissions: fluoroquinolone monotherapy (moxifloxacin) and a macrolide plus cephalosporin combination (Azithromycin plus Ceftriaxone). It was found that the use of the macrolide plus cephalosporin combination had strong value in the prediction of COPD readmissions. Adams et al. (35) found that use of macrolide and cephalosporin antibiotics were associated with the lowest COPD readmission rates, after comparing numerous antibiotics. It is probable that this relationship holds true in the present study.

In assessing the strengths and weaknesses of this study, one of its major strengths is that its mean AUC of 0.75 suggests a high level of model accuracy. To place this AUC in perspective, the Model of End-Stage Liver Disease (MELD) score, a commonly used prediction tool that predicts 90-daymortality for patients with end-stage liver disease, has been reported in literature to have an AUC ranging anywhere from 0.7 to 0.8 (39–41). Furthermore, this study has a high level of reliability by virtue of the analysis algorithm creating 1,000 different regression trees for each data run, with each tree being a voting contributor to the sensitivity analysis. In addition, 200 runs of the prediction model were completed to ensure the results had a reliable and reproducible output. In addition, we considered 55 variables, all of which have been found to be highly associated with COPD readmission, for our initial analysis. This broad consideration of risk factors surely would lend itself to a comprehensive analysis. Thus, another strength of our study is its utility; any risk stratification algorithm generated would be predicated on the five variables discussed earlier, making it easy to manipulate in making patient-care decisions.

As with all research, our study does have some limitations. First, data from only 106 patients were used in creating the prediction model. However, one of the strengths inherent in the RF model statistical design is that it is built to analyze complex problems with small samples by manipulating a large number of variables (11, 12). Furthermore, this study was also conducted in only two academic community hospitals in central Pennsylvania. Therefore, to further validate this prediction model, we recommend that our generated model be tested in other venues.

Another perceived weakness that deserves review is the lack of directionality of the associations made by the RF prediction model. Previous studies have already validated the direction of association of these risk factors with the outcome of interest. Thus, the results of this study are actually valuable when taken in the context of previous literature. For example, previous literature has already shown that both low and high BMI are associated with increased COPD exacerbations. Our study shows that when collecting BMI with four other specific critical variables and entering the data into the regression tree analysis that makes up the RF prediction algorithm, certain combinations of the values of these five variables will be able to predict the probability of that patient being readmitted within a single year. In this lies the innovative nature of this prediction algorithm.

The algorithm/equation produced in this study is not the standard algorithm seen elsewhere, such as in the form: A + B+C + D+E = F. It is composed of a series of mathematical regression trees that are not reproducible on a flow diagram but can be recreated using the ‘coding’ from the RF computer system. This study's model was in fact composed of 1,000 regression trees, with each one being a voting contributor to the prediction model. Each tree was partitioned into a ‘readmission arm’ and a ‘non-readmission arm’ based on the validation training sets (multiple validation sets were run as discussed earlier). Depending on which arm of each tree the patient's data fell under determined the type of vote that tree would provide, with the sum of the 1,000 votes creating the predicted outcome. This prediction algorithm is ‘encoded’ in the mathematical script from the RF computer software developed in this study.

The results of this study indicate that hospitals need to focus on COPD patients from the time of admission, rather than simply at the time of discharge. It should begin with better nutrition in the hospital, weight-loss counseling, more aggressive postoperative pulmonary rehabilitation, social work for job placement, and organization of more regular follow-up appointments. The creation of a COPD specialty clinic for more aggressive disease management is also a logical solution to ensure that the therapeutic interventions begun during the hospital admission are continued once the patient is discharged. Literature has consistently shown that heart failure clinics have reduced mortality and readmission rates for patients with congestive heart failure; therefore, it is reasonable to expect similar results from a COPD specialty clinic (42, 43).

Undergoing a COPD exacerbation has been likened to being smothered. Lack of education, or a system that has long neglected COPD patients, calls for further research on new approaches to identify and treat high-risk COPD patients. Whether from an economic point-of-view with the potential to save millions of dollars, or from a patient-centered focus with the potential to decrease death and disability, the RF prediction model, and other artificial intelligence models like it, may provide us with a more effective way to deploy our limited health care resources. Further research on the particulars of COPD exacerbations is clearly required and validation of our statistical model is undoubtedly warranted in other venues and with other populations. Given the established robustness of the RF methodology, the results of our study suggest that the medical community now has the available tools to tame the public health scourge, that is, COPD.
